# Whole genome sequence data of a marine bacterium*, Marinobacter adhaerens* PBVC038, associated with toxic harmful algal bloom

**DOI:** 10.1016/j.dib.2022.108768

**Published:** 2022-12-05

**Authors:** Grace Joy Wei Lie Chin, Jaeyres Jani, Salley Venda Law, Kenneth Francis Rodrigues

**Affiliations:** a Biotechnology Research Institute, Universiti Malaysia Sabah, Jalan UMS, Kota Kinabalu, Sabah 88400, Malaysia; b Borneo Medical and Health Research Centre, Faculty of Medicine and Health Science, Universiti Malaysia Sabah, Jalan UMS, Kota Kinabalu, Sabah 88400, Malaysia

**Keywords:** Associated bacteria, Harmful algal bloom, Microbial genomics, Paralytic shellfish toxin, *Pyrodinium bahamense* var. *compressum*

## Abstract

*Marinobacter adhaerens* (PBVC038) was isolated from a harmful algal bloom event caused by the toxic dinoflagellate *Pyrodinium bahamense* var. *compressum* (*P. bahamense*) in Sepanggar Bay, Sabah, Malaysia, in December 2012. Blooms of *P. bahamense* are frequently linked to paralytic shellfish poisoning, resulting in morbidity and mortality. Prior experimental evidence has implicated the role of symbiotic bacteria in bloom dynamics and the synthesis of biotoxins. The draft genome sequence data of a harmful algal bloom-associated bacterium, *Marinobacter adhaerens* PBVC038 is presented here. The genome is made up of 21 contigs with an estimated 4*,*246*,*508 bases in genome size and a GC content of 57.19%. The raw data files can be retrieved from the National Center for Biotechnology Information (NCBI) under the Bioproject number PRJNA320140. The assessment of bacterial communities associated with harmful algal bloom should be studied more extensively as more data is needed to ascertain the functions of these associated bacteria during a bloom event.

**Specifications Table**

**Table:** 

Subject	Marine Microbiology
Specific subject area	Genomics and Bioinformatics
Type of data	Genome sequence data, table and figure
How the data were acquired	Microscope imaging: Scanning Electron Microscope (SEM) Whole-genome sequencing: Illumina MiSeq Data analysis: Fastx toolkit, Scythe v0.994, Trimmomatic v0.35, Iterative *De Brujin* Graph *De Novo* Assembler IDBA-UD software, CheckM, rapid prokaryotic genome annotation (PROKKA), and Rapid Annotation using Subsystem Technology (RAST) v2.0, Roary v3.11.2, MEGAX.
Data format	Raw and analyzed
Description of data collection	The reads of *Marinobacter adhaerens* PBVC038 was produced from MiSeq platform and assembled using Iterative *De Brujin* Graph *De Novo* Assembler IDBA-UD software. The genome annotation was accomplished using PROKKA and RAST.
Data source location	Biotechnology Research Institute, Universiti Malaysia Sabah, Kota Kinabalu, Sabah, Malaysia.
Data accessibility	The sequencing data [1] is available in the NCBI repository: Bioproject: PRJNA320140 (https://www.ncbi.nlm.nih.gov/bioproject/PRJNA320140) NCBI Genbank accession number: LXRF00000000 (https://www.ncbi.nlm.nih.gov/nuccore/LXRF00000000) NCBI SRA accession number: SRX1737533 (https://www.ncbi.nlm.nih.gov/sra/SRX1737533)

**Value of the Data**

• The genome analysis showed the presence of *Marinobacter adhaerens* strain PBVC038 in a toxic algal bloom associated with the *P. bahamense* in Sepanggar Bay, Kota Kinabalu, Sabah.
• The dataset aids in understanding the function of an associated bacterium, *Marinobacter adhaerens*, during a toxic bloom, by analyzing the genes associated with bloom dynamics and the biosynthesis of toxins.
• Marine researchers can use the data to compare with the genomes of other harmful algal bloom-associated bacteria.
• Researchers could use the genome information provided here to comprehend the genetic-related mechanisms of a marine bacterium responsible for harmful algal bloom and toxin production.

## 1 Objective

The primary goal of generating this dataset was to study how the associated bacteria contribute to bloom dynamics and the production of biotoxins based on next-generation sequencing. Previous studies on the associated bacteria of the same harmful marine microalga, *P. bahamense* var. *compressum*, used the 16S rRNA gene as a DNA barcoding marker and 16S metagenome sequencing to study the associated bacterial community. The studies uncovered several intriguing bacterial species previously reported to be associated with toxic, harmful algal bloom and involved directly and indirectly with toxin production. Hence, this genome dataset was acquired to analyze the genes associated with bloom dynamics and toxin biosynthesis comprehensively.

## 2 Data Description

**Fig. 1:**
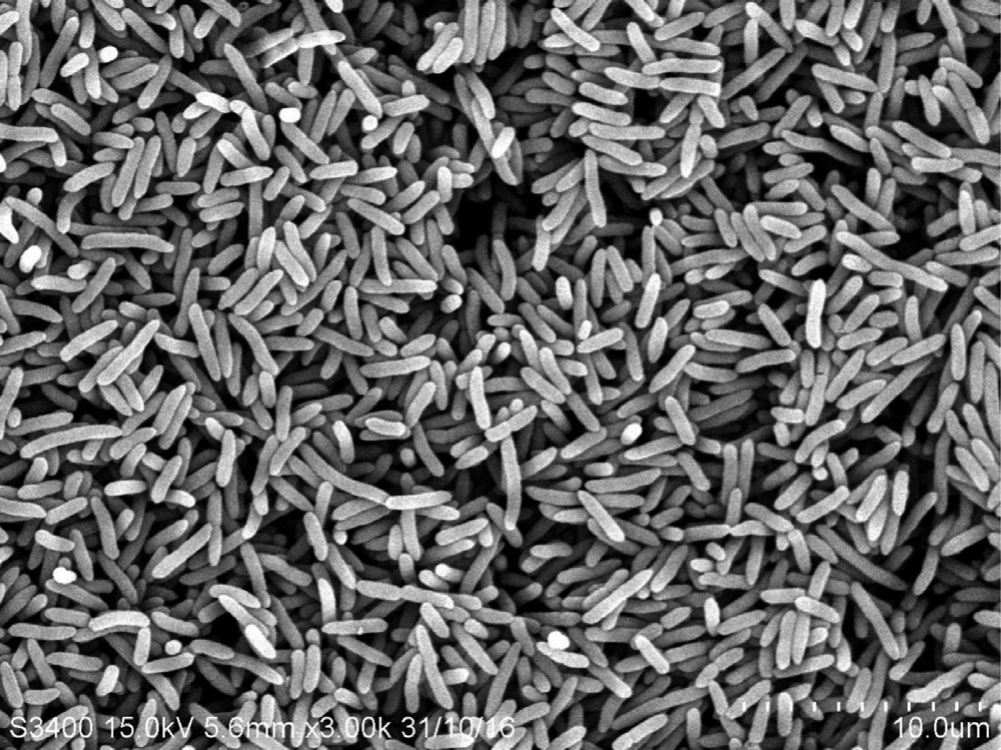
Scanning electron microscope image of *Marinobacter adhaerens* PBVC038.

**Fig. 2:**
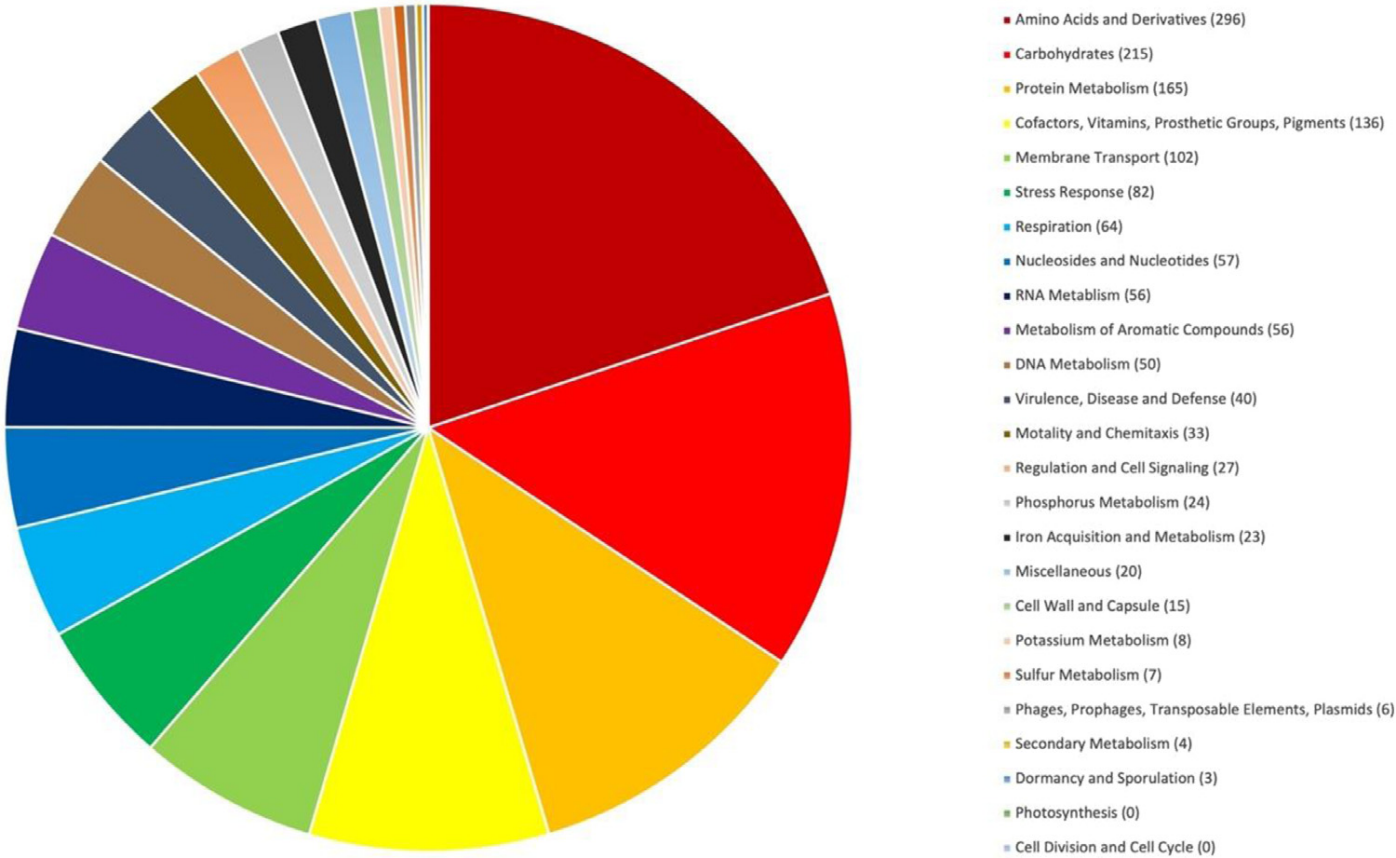
RAST server's output summary of functional distribution of protein-coding genes in the *Marinobacter adhaerens* PBVC038.

**Fig. 3:**
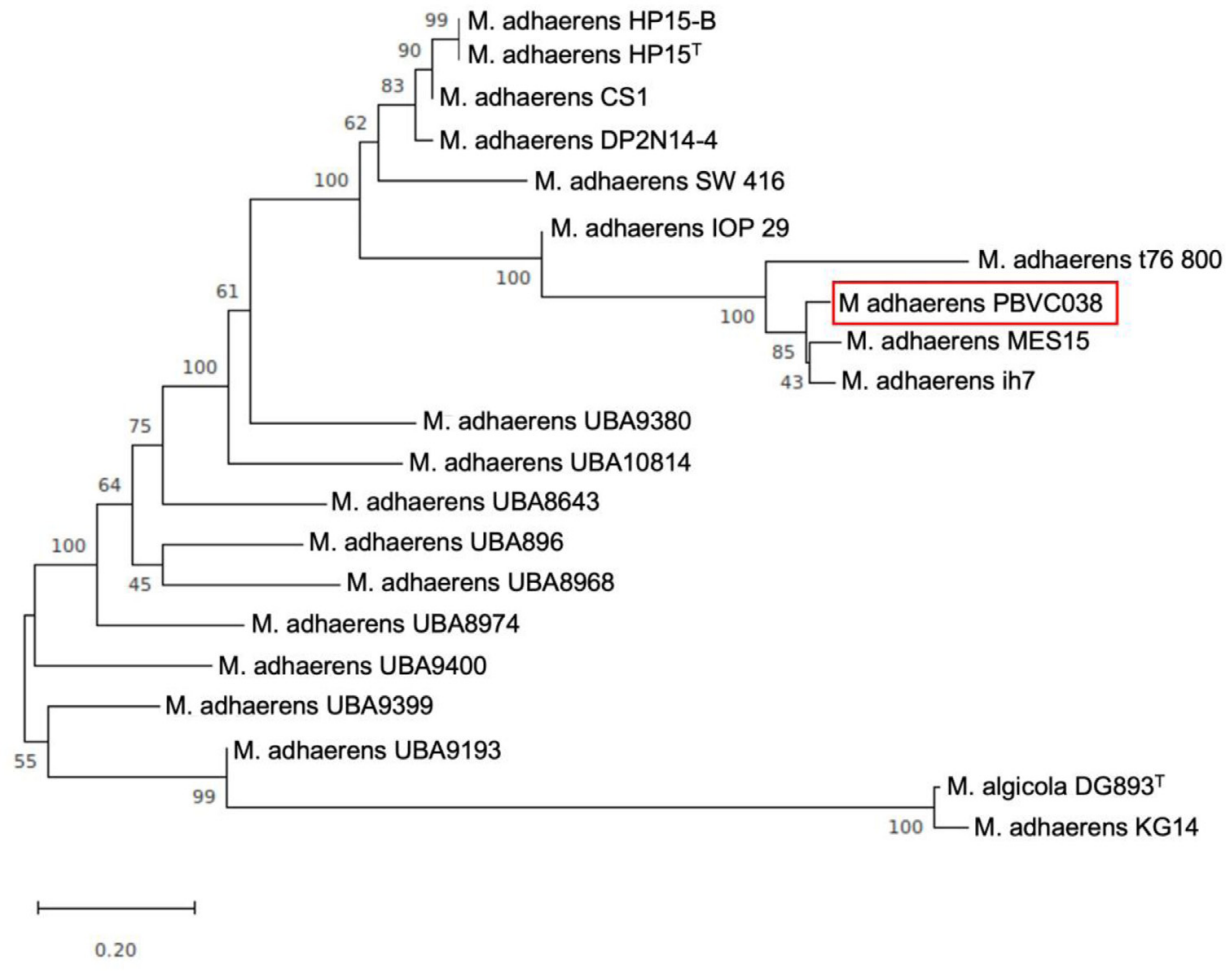
Phylogenetic tree showing the position of *Marinobacter adhaerens* PBVC038 (highlighted in red box) based on the core genome sequences in relative to 19 other genomes of *Marinobacter adhaerens* from the NCBI database. Superscript ‘T’ indicates type strain. Bootstrap values of 1000 replicates are reported above all nodes.

A harmful algal bloom of the toxic microalga, *P. bahamense*, occurred in the coastal waters of west Sabah, Malaysia, in December 2012. Following the detection of the toxic bloom in the coastal waters, the state Department of Fisheries issued a warning to the public to prevent them from consuming any shellfish or bivalves harvested from the affected areas. The shellfish samples collected from the affected regions have toxin levels as high as 4010 Mouse Units (MU), where 400 MU is considered the lowest amount harmful to humans [2]. We isolated a rod-shaped marine bacterium, *Marinobacter adhaerens* PBVC038 (Fig. 1), from the harmful alga, *P. bahamense*, and confirmed its identity through 16S rRNA Sanger sequencing [3]. Bacteria from the genus *Marinobacter* are the largest genus in the family Marinobacteraceae, and are frequently reported to be associated with harmful algal bloom [4]. Next, we performed whole genome sequencing of the associated bacterium. The whole-genome sequencing generated approximately 593.3 Mb total bases, totaling about 1,203,928 raw pair-end reads. The draft genome has a total size of 4,246,508 bp after assembly, with 21 contigs, a GC% of 57.2, and an N50 score of 1,000,299 bp. The sequencing coverage was 100×. CheckM analysis confirmed that the genome has 100% completeness and 0% contamination. The graphical representation in Fig. 2 summarizes the functional annotation of the *M. adhaerens* PBVC038 genome, where 3891 protein-coding genes were identified (Table 1). A core genome phylogenetic tree was constructed using PBVC038 and a group of *M. adhaerens* genomes retrieved from the NCBI databases (Fig. 3). The phylogeny showed that *M. adhaerens* PBVC038 was grouped with the Brazilian *M. adhaerens* t76_800, Saudi Arabian *M. adhaerens* MES15 and American *M. adhaerens* ih7, with the bootstrap value of 85–100%. The strain PBVC038 had an average nucleotide identity (ANI) of more than 95% with its closely related *M. adhaerens* strains t76_800, MES15, and ih7 (Table 2).

**Table 1:** Statistics of the whole-genome data of *Marinobacter adhaerens* PBVC038.

Raw data	1203,928 reads (593.3 M bases)
Sequencing coverage	100×
No. of contigs	21
Genome length	4246,508 bp
N50	1000,299 bp
GC content	57.19%
Total genes	3891
Number of tRNA, rRNA, ncRN	49, 10, 4

**Table 2:** Average Nucleotide Identity (ANI) values of *Marinobacter adhaerens* PBVC038 with five other *Marinobacter* strains.

	PBVC038	MES15	ih7	t76_800	HP15	DG893
PBVC038		96.49	96.95	95.17	87.07	76.19
MES15	96.49		96.29	94.30	86.48	75.97
ih7	96.95	96.29		95.31	86.36	75.74
t76_800	95.17	94.30	95.31		89.95	75.41
HP15	87.07	86.48	86.36	89.95		76.20
DG893	76.19	75.97	75.74	75.41	76.20	

## 3 Experimental Design, Materials, and Methods

### 3.1 Study Area

The bacterium was isolated from Sepanggar Bay, Sabah, Malaysia, one of the affected regions impacted by the 2012 harmful algal bloom. During the sample period, high paralytic shellfish toxin or saxitoxin levels of up to 3300 Mouse Units were detected [5,6].

### 3.2 Isolation of Bacterium *M. adhaerens* PBVC038

Seawater samples from the bloom areas were collected using a plankton net of 20 µm mesh size and stored in sterile sampling bottles. Single cells of *P. bahamense* were isolated from the newly acquired seawater samples under a light microscope using the drawn-out micropipettes [7]. The unialgal culture of *P. bahamense* was established under the following conditions: the temperature at 28 °C in a 12-hour shift of light-dark cycle and light intensity of 150 µEm^-^^2^s^−1^. A total of 1 ml of *P. bahamense* culture at mid-exponential growth phase was aliquot onto a sterile marine agar (Difco) and incubated for at least 72 h at 37 °C. Once the bacteria had grown, the individual bacterial colonies were sub-cultured onto a fresh, sterile marine agar plate. The bacterium PBVC038 was effectively recovered from the agar plate after an overnight 37 °C incubation period.

### 3.3 Scanning Electron Microscopy (SEM)

In order to prepare for SEM, the bacterium was cultivated in marine broth (Difco) and agitated at 200 rpm in an orbital shaker, with an incubation temperature of 25 °C. The bacterial cells were collected by centrifuging at 11,000 × *g* for 15 min after two days of incubation. The sample preparation for SEM was carried out according to Yik et al. [8]. The bacterial sample was fixed in a solution of 5% glutaraldehyde and 0.1 M phosphate buffer (pH 7.2), which was then left to sit at 4 °C for 24 h. The sample was washed with phosphate buffer before being gradually dehydrated in 35, 50, 75, and 95% ethanol solutions. Next, absolute ethanol was used twice to complete the dehydration process. Each ethanol solution wash step took five minutes to complete. The sample was mounted onto an SEM specimen stub and covered in gold after being air dried in a desiccator at room temperature for 16 h. The sample was viewed with a Hitachi S-3400 N scanning electron microscope.

### 3.4 DNA Extraction

For DNA extraction, the overnight (15–16 h) bacterial cell culture was pooled by centrifuging at 5000 × g for 10 min. The DNA extraction method was carried out according to protocols described in the Qiagen DNeasy Blood and Tissue Kit manual (Qiagen Biotechnology). The quality of the extracted DNA was examined using the Qubit dsDNA HS Assay kit (Thermo Fisher Scientific).

### 3.5 Genome Sequencing, Assembly, and Annotation

A standard genomic 250 bp paired-end library was produced after DNA extraction using the Illumina Nextera XT DNA Library Preparation Kit. Libraries were quantified and normalized to 4 nM before pooling and sequencing the libraries using the Illumina MiSeq system. Quality filtering of the acquired sequencing reads was conducted using the Fastq Quality Filter in Fastx Toolkit [9]. The adapter sequence was removed using Scythe v0.994 [10], whereas the trimming of the reads based on a Q trimming threshold of 25 was performed using Trimmomatic v0.35 [11]. Iterative *De Brujin* Graph *De Novo* Assembler IDBA-UD software was used to *de novo* assemble the trimmed reads [12], and the completeness of the genome was analyzed using CheckM [13]. The functional annotations were performed using PROKKA [14] and the RAST server [15].

### 3.6 Phylogenetic Tree Analysis

A total of 21 genomes of *Marinobacter* strains were subjected to pan-genome analysis using Roary v3.11.2 [16]. The input files used were the GFF3 (format generated from the annotated assembly (Prodigal) of *Marinobacter adhaerens* PBVC038) and GenBank files obtained from the NCBI website (20 strains). The threshold of 99% was used to determine core genes across all strains, whereas BLASTP was used to compare the sequences of different strains using a user-defined percentage of sequence identity of 95% [17]. The division of homologous groups comprising paralogs into groups of true orthologs was carried out using data on conserved gene neighbourhoods. Gene presence in the accessory genome was used to group the strains together, which was weighted by the total and shared genomes. A core-genome phylogenetic tree was constructed using the maximum likelihood method in MEGAX [18] with a bootstrap analysis of 1000 replicates. Average nucleotide identities were calculated using ANI calculator (http://enve-omics.ce.gatech.edu/ani/) [19].

## Ethics Statement

Since the work provided here did not involve employing human participants, animal trials, or data obtained from social media platforms, no ethical statements were required for this data.

## CRediT authorship contribution statement

**Grace Joy Wei Lie Chin:** Conceptualization, Funding acquisition, Supervision, Writing – original draft. **Jaeyres Jani:** Software, Formal analysis, Data curation, Writing – review & editing. **Salley Venda Law:** Resources, Investigation. **Kenneth Francis Rodrigues:** Methodology, Supervision, Validation, Writing – review & editing.

## Declaration of Competing Interest

The authors declare that they have no known competing financial interests or personal relationships that could have appeared to influence the work reported in this paper.

## Data Availability

Draft Genome Sequence of Marinobacter adhaerens, strain PBVC038 (Original data) (Sequence Read Archive (SRA) of National Center of Biotechnology Information).
